# Pilot Evaluation of Two *Fasciola hepatica* Biomarkers for Supporting Triclabendazole (TCBZ) Efficacy Diagnostics

**DOI:** 10.3390/molecules25153477

**Published:** 2020-07-30

**Authors:** Clare F. Collett, Russell M. Morphew, David Timson, Helen C. Phillips, Peter M. Brophy

**Affiliations:** 1 Institute of Biological, Environmental and Rural Sciences, Aberystwyth University, Aberystwyth SY23 3DA, UK; rom@aber.ac.uk (R.M.M.); hcp5@aber.ac.uk (H.C.P.); pmb@aber.ac.uk (P.M.B.); 2 School of Pharmacy and Biomolecular Sciences, University of Brighton, Brighton BN2 4GJ, UK; d.timson@brighton.ac.uk

**Keywords:** fasciolosis, triclabendazole, diagnostics, biomarker, calreticulin, triose phosphate isomerase

## Abstract

*Fasciola hepatica*, the causative agent of fasciolosis, is a global threat to public health, animal welfare, agricultural productivity, and food security. In the ongoing absence of a commercial vaccine, independent emergences of anthelmintic-resistant parasite populations worldwide are threatening the sustainability of the few flukicides presently available, and particularly triclabendazole (TCBZ) as the drug of choice. Consequently, prognoses for future fasciolosis control and sustained TCBZ application necessitate improvements in diagnostic tools to identify anthelmintic efficacy. Previously, we have shown that proteomic fingerprinting of *F. hepatica* excretory/secretory (ES) products offered new biomarkers associated with in vitro TCBZ-sulfoxide (SO) recovery or death. In the current paper, two of these biomarkers (calreticulin (CRT) and triose phosphate isomerase (TPI)) were recombinantly expressed and evaluated to measure TCBZ efficacy via a novel approach to decipher fluke molecular phenotypes independently of molecular parasite resistance mechanism(s), which are still not fully characterised or understood. Our findings confirmed the immunoreactivity and diagnostic potential of the present target antigens by sera from TCBZ-susceptible (TCBZ-S) and TCBZ-resistant (TCBZ-R) *F. hepatica* experimentally infected sheep.

## 1. Introduction

*Fasciola hepatica*, the common liver fluke and causative agent of fasciolosis, is a major zoonotic parasitic platyhelminth of humans and their livestock within temperate regions worldwide. Liver fluke threatens food security via acute infections, resulting in sudden flock deaths, as well as chronic infections leading to reductions in meat and/or milk yields, fertility, and/or wool production, which together contribute to annual global economic losses estimated to be over $3BN [1,2]. *F. hepatica* is also a re-emerging food-borne disease risk to human populations, with up to 17 million people estimated with infection and 180,000 at risk [3]. Meteorological trends associated with climate change and global warming are expected to increase the geographic spread of this parasite, and its intermediate Lymnaeid snail host [3], factors that are favourable for perpetual disease transmission. Thus, these climatological and biological shifts are predicted to prompt epidemic livestock disease incidence rates in years to come [4] and further increase public health concerns.

Triclabendazole (TCBZ) and its metabolites (−SO/−SO_2_) comprise the only multi-stage flukicide agent that targets all mammalian-infective immature and mature fluke stages with high efficacy and broad activity [5]. Consequently, overreliance has subsequently led to global reports of TCBZ resistance (TCBZ-R) and treatment failure in both livestock and humans [6,7]. New tools that can determine TCBZ efficacy status are urgently needed to support TCBZ management in the absence of vaccines and new efficacious anthelmintics [8], knowledge that will inform treatment selection and likely prolong TCBZ shelf-life. Currently, there are no biomarkers directly associated with *F. hepatica* TCBZ efficacy, as the majority of diagnostic groundwork and method development has been aimed towards parasite detection per se and through subsequent reduction tests, for example, based on the recovery of eggs and the detection of *F. hepatica* faecal antigens such as cathepsin L (CL) proteases or other excretory/secretory (ES) proteins [9,10,11]. However, we have previously shown that proteomic fingerprinting of *F. hepatica* ES products offers new biomarkers associated with TCBZ-sulfoxide (SO) recovery or death [11]. In the present study, two of these biomarkers, calreticulin (CRT) and triose phosphate isomerase (TPI), were recombinantly expressed and evaluated using experimental infection sera as candidates of a novel molecular test platform to measure TCBZ efficacy.

## 2. Results

### 2.1. In Vitro Proteomics: Recombinant Expression and Purification

#### 2.1.1. Calreticulin (*N*-Terminally Truncated, rFh∆CRT)

**Figure 1:**
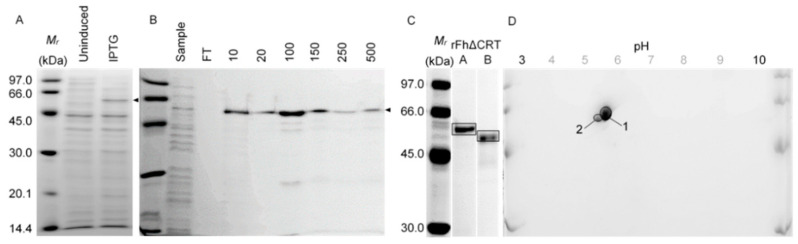
Expression, purification and proteomic analysis of recombinant truncated *Fasciola hepatica* calreticulin (rFhΔCRT). *FhΔCRT*-pET-28b(+) BL21(DE3)pLysS lysates (5 µg) before and after 0.5 mM isopropyl β-d-1–thiogalactopyranoside (IPTG) induction were analysed by 1-DE (**A**) before immobilised metal ion chromatography (IMAC) purification using HIS-Select^®^ Cobalt resin (**B**) over two operations conducted at pH 6.0, followed by further purification at pH 8.0 (not shown), to remove co-elutants with the target rFh∆CRT, indicated by the arrow. Transient dimorphism of rFh∆CRT at ~48 and ~50 kDa was evident following re-purification, owing to the pH adjustment ((**C**), samples (**A**) vs. (**B**), 1.5 µg per lane). 2-DE of the nominal ~50 kDa purified sample ((**D**), 5 µg) (boxed/encircled) demonstrated further isomeric separation of this rFh∆CRT between approximately 5.25 and 5.50 pH, which was higher than predicted (predicted: 4.79 pI including polyhistidine tag). Both dimorphic (1-DE, boxed) and isomeric (2-DE, encircled) proteins were confirmed as F. hepatica calreticulin by LC-MS/MS (*p* < 0.01: PRJEB25283, maker-scaffold10x_1445_pilon-snap-gene-0.69.mRNA-1). Abbreviations: *M_r_*, Amersham Low Molecular Weight sodium dodecyl sulfate (SDS) Calibration Kit (kilodaltons); FT, flow-through; 10–500 [mM] imidazole concentration.

rFh∆CRT was overexpressed using 0.5 mM isopropyl β-d-1–thiogalactopyranoside (IPTG) (Figure 1A, 54.9 kDa) and purified using immobilised metal ion chromatography (IMAC) (Figure 1B). Initially, IMAC at sub-neutral pH (pH 6) was attempted, but subsequently superseded by above neutral pH (pH 8). Isomeric separation of rFh∆CRT was noticeable and shown by 1-DE (pH-associated: Figure 1A, at pH 6; Figure 1B at pH 8) and 2-DE (Figure 1D), which further demonstrated isoelectric separation of these components between approximately 5.25 and 5.50 pI. Molecular weight and pI properties were also augmented compared with predicted values for Fh∆CRT of 46.99 kDa (+1.28 kDa including polyhisitidine tag) and 4.67 pI (+0.12 pI including polyhisitidine tag), though dimorphism was reminiscent of previous descriptions of *Trypanosoma cruzi* CRT [12].

**Figure A1:**
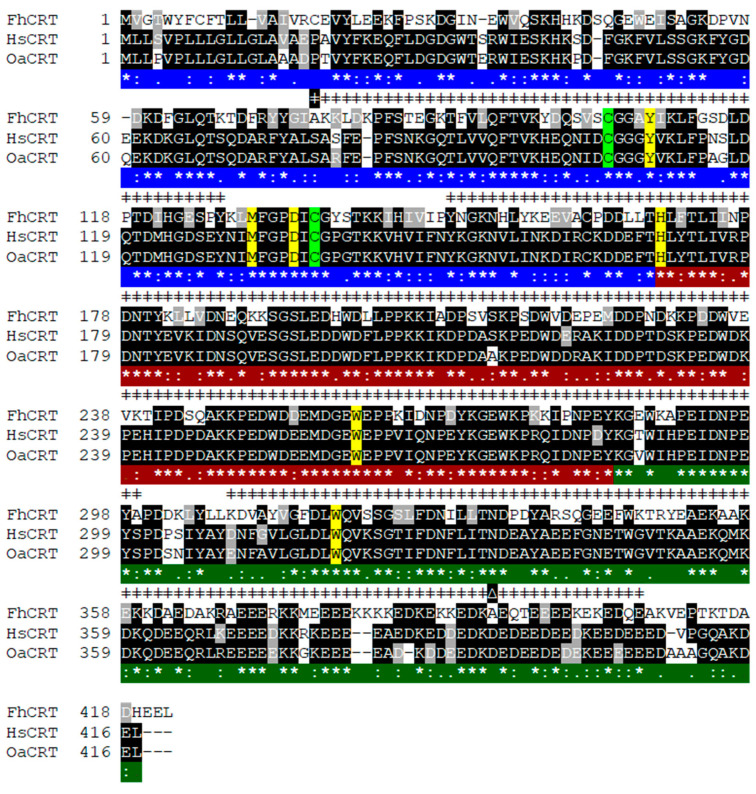
LC-MS/MS identification of FhCRT and comparison with host CRT proteins via sequence alignment. Sequence analysis of FhCRT is shown, including LC-MS/MS-identified residues (ǂ: PRJEB6687, BN1106_s2673B000071.mRNA-1/PRJEB25283, maker-scaffold10x_1445_pilon-snap-gene-0.69.mRNA-1) based on two 2-DE-separated isomers and two dimorphic 1-DE-separated proteins (Figure 1C,D), which identified 71.8% residues of the complete sequence and 75.3% residues when excluding the truncated signal peptide (1–21 aa). Peptide fragmentation of the highest MASCOT scoring sample (Figure 1D: major spot) indicated a putative aspartate-asparagine substitution (residues above ∆: FhCRT, D333N; Fh∆CRT, D316N). Alignment of FhCRT with human (P27797) and ovine (W5PJG7) host proteins was conducted to identify sequence identity of 51.9% and 51.4%, respectively. On the basis of studies of human and mouse CRTs [51], N (1–169 aa), P (170–284 aa), and C (285–400 aa) calreticulin domains are shown, and further residues of known functional (as highlighted: Y-109, M-131, D135, H-170, W261, W-319) and both structural and functional (as highlighted: C-105, C-137) importance were fully conserved in FhCRT and OaCRT. Abbreviations: *, conserved; strongly semi-conserved; weakly semi-conserved.

LC-MS/MS identified 1-DE- and 2-DE-separated rFh∆CRT isomers (Figure 1C,D: boxed/circled protein bands/spots) as FhCRT (WormBase ParaSite (WBPS): PRJEB25283, maker-scaffold10x_1445_pilon-snap-gene-0.69.mRNA-1). Specifically, >71.8% of all FhCRT residues were detected (**ǂ**, Figure A1), which, when excluding the truncated signal peptide (1–19 aa, not including Cys-18), increased to >75.3% overall sequence coverage. On the basis of representative Sanger Sequencing data (not shown), a putative aspartate-asparagine substitution was determined (Figure A1: FhCRT, D333N; Fh∆CRT, D316N). This was supported by peptide fragmentation analysis in the highest MASCOT scoring LC-MS/MS submission (Figure 1D: spot 1; 1842 MASCOT score), within the MS/MS-derived peptide D309-R339 from FhCRT (1-DVAYVGFDLWQVSSGSLFDNILLTDDPDYAR-31, 46.77% D25N verses 27.79% D19N; 14.32% D26N; 11.12% D28N). Interestingly, this sequence change is different from another FhCRT genome project sequence (Genbank: PIS80991.1; not shown), but is conserved between human (HsCRT) and ovine (OaCRT) host CRTs, sharing 51.9% and 51.4% overall sequence identity with FhCRT, respectively. On the basis of sequence matching to HsCRT (Figure A1), mouse, and rabbit CRT models [13,14,15] (not shown), inferences were drawn for domain allocations, including lectin (N, 1–169 aa), proline-rich (P, 170–284 aa), and C-terminal (C, 285–400 aa) CRT domains (Figure A1). Further residues of reported biological importance in mouse and rabbit CRTs that were fully conserved here (Figure A1) included N-, P-, and C-based residues towards ER-based glycan interactions, protein binding, and folding (FhCRT, Y-109, M-131, D-135, H-170, W-261, W-319), and those involved in the formation of a disulfide bond within the N domain (FhCRT, C-105, C-137).

#### 2.1.2. Triose Phosphate Isomerase (rFhTPI)

**Figure 2:**
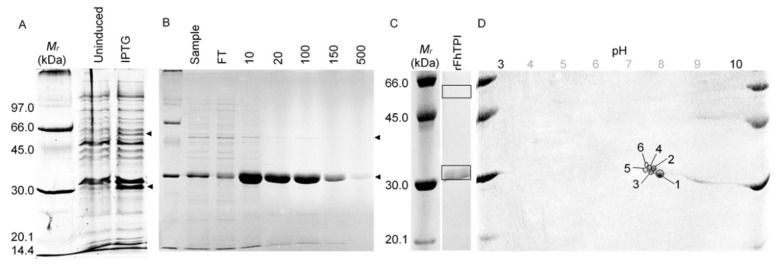
Expression, purification, and proteomic analysis of recombinant *Fasciola hepatica* triose phosphate isomerase (FhTPI). *FhTPI*-pET-46Ek/LIC One Shot^®^ BL21(DE3) lysates (5 µg) before and after 0.5 mM IPTG induction were analysed by 1-DE (**A**) before IMAC purification using HIS-Select^®^ Nickel resin (**B**), with rFhTPI indicated by the arrows. Then, 1 μg purified rFhTPI was analysed by 1-DE (**C**) and 2-DE (**D**) (boxed/encircled), showing a single major band at approximately 30 kDa (predicted: 29.49 kDa including polyhistidine tag), but six defined isomer spots within the region of 7.00–7.60 pH, which was slightly higher than expected (predicted: 6.89 pI including polyhistidine tag). LC-MS/MS identified all isomers (including duplicate submissions of 1-DE separated monomer and dimer) as *F. hepatica* triose phosphate isomerase (*p* < 0.01: GenBank, AGJ83762.1). Abbreviations: *M_r_*, Amersham Low Molecular Weight SDS Calibration Kit (kilodaltons); FT, flow-through; 10–500 [mM] imidazole concentration.

rFhTPI was overexpressed using 0.5 mM IPTG (Figure 2A, 29.7 kDa) and purified using IMAC (Figure 2B). 1-DE indicated the majority of protein resolution was monomeric and with minor dimerization (Figure 2C), likely owing to the absence of cross-linkers [16]. 2-DE demonstrated six rFhTPI isoforms separated between approximately 7.50 and 8.10 pI (Figure 2D), properties that were higher than the predicted values for rFhTPI of 27.78 kDa (+1.71 kDa including polyhistidine tag) and 8.07 pI (−1.18 pI including polyhistidine tag).

LC-MS/MS identified 1-DE- and 2-DE-separated rFhTPI isomers (Figure 2C,D: boxed/circled) as FhTPI (GenBank, AGJ83762.1), including >86.5% of FhTPI residues (**ǂ**, Figure 2A). The comparison of FhTPI to host TPIs, including HsTPI and OaTPI, demonstrated 68.6% ID with both sequences and allowed matching of residues of putative structural and functional importance. The highly conserved TPI active site is putatively shown (Figure 2A: FhTPI, A-167-T-176), and the residues involved in dimerization (FhTPI, T-76, E-98), catalysis (FhTPI, K-14), or both processes (FhTPI, H-86, E-169) are highlighted based on biochemical studies of human and other model TPIs [17,18,19,20], whereby all residues of functional and structural importance were shown between the three sequences studied here.

### 2.2. Western Hybridisation

**Figure 3:**
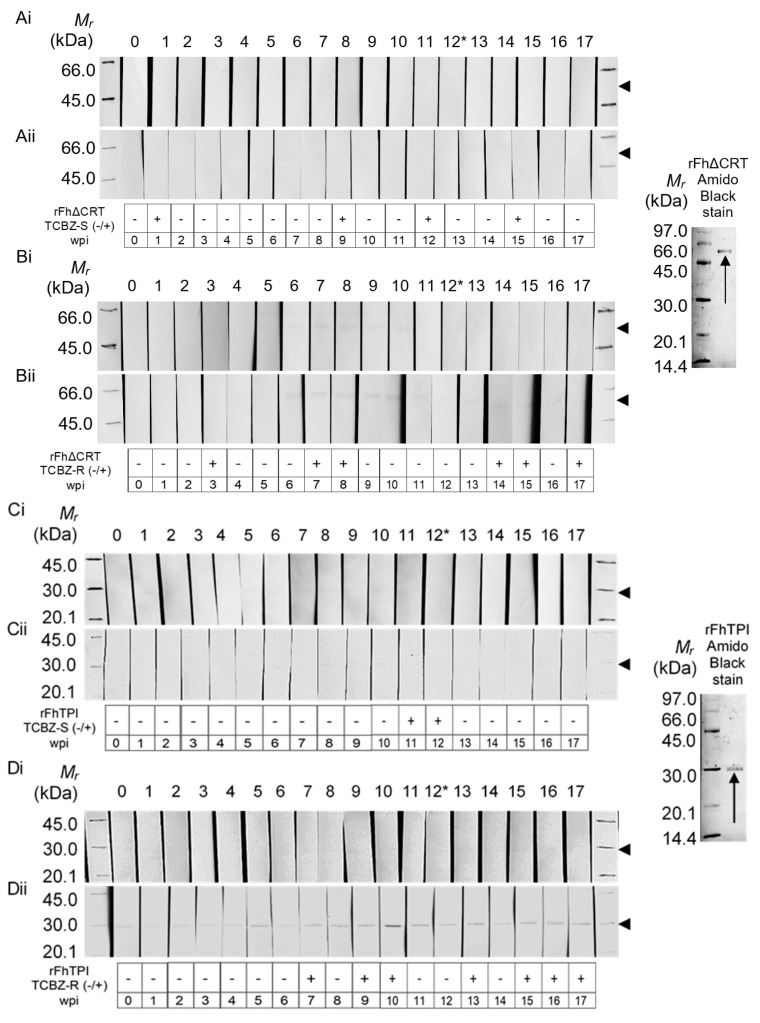
Quantitative representation of duplicate western hybridisations for recombinant biomarker immunogenicity testing against triclabendazole-susceptible (TCBZ-S) and TCBZ-resistant (TCBZ-R) *Fasciola hepatica*-infected sheep IgG. rFhΔCRT (0.25 μg/lane) (**A**,**B**) and rFhTPI (0.25 μg/lane) (**C**,**D**) were probed with whole sheep sera diluted to 1:1500 and pooled from three animals experimentally infected with TCBZ-susceptible (TCBZ-S; **A**,**C**) or TCBZ-resistant (TCBZ-R; **B**,**D**) *F. hepatica* isolates, and subsequently detected for IgG binding via the 5–bromo-4–chloro-3–indolyl-phosphate and nitro blue tetrazolium (BCIP/NBT) system. Data from duplicate western hybridisations were analysed (**i**–**ii**) and band areas of interest were quantified using ImageQuant TL (GE Healthcare Life Sciences, UK), which calculated positivity (+) or negativity (−) by subtracting 0 weeks post infection (wpi) values from each wpi value for each treatment, whereby values that were negative, or negative and positive in both replicates, were considered negative. Nitrocellulose membrane (NCM) transfer of 1-DE-separated recombinant proteins was confirmed using Amido Black staining. Abbreviations: *M_r_*, Amersham Low Molecular Weight SDS Calibration Kit (kilodaltons); 0–17, weeks post-infection; *, TCBZ administration.

Immunodiagnostic evaluations of rFh∆CRT and rFhTPI were conducted via western hybridisation with 0.25 μg/lane. Antigens were probed with serum pooled from experimental infections (day 0–119; 0–17 weeks post infection (wpi)) in sheep (dosed with 250 metacercariae), with one each of three TCBZ-susceptible (TCBZ-S) (Aberystwyth, Italian, Miskin) or TCBZ-R (Kilmarnock, Penrith, Stornoway) *F. hepatica* strains. Whole sera were probed and IgG binding was detected using the 5–bromo-4–chloro-3–indolyl-phosphate and nitro blue tetrazolium (BCIP/NBT) system (Figure 3). To improve the sensitivity of immunoreactivity for interpretation, bands were quantified using ImageQuant TL (GE Healthcare Life Sciences, UK) via band intensity values, whereby 0 wpi values were subtracted from 1 to 17 wpi values per blot and averaged between duplicate western hybridisations to determine the overall immunoreactivity (negative, −; positive, +).

Blots indicated differential immunoreactivity between candidates, indicating *F. hepatica* TCBZ efficacy-dependent differences and temporal changes in native antigen abundance and exposure post-infection, reflected by IgG reactivity to recombinant biomarkers (Figure 3). TCBZ-S-specific sera demonstrated antibody-rFhΔCRT binding at 1, 9, 12, and 15 wpi (Figure 3A), whereas TCBZ-R-specific sera indicated rFhΔCRT recognition at 3, 7–8, 14–15, and 17 wpi (Figure 3B). rFhTPI indicated limited immunoreactivity with both sets of sera, though positivity was evident first in TCBZ-R infection at 7, 9, 10, 13, and 15–17 wpi (Figure 3D) compared with TCBZ-S-specific sera positivity at only 11–12 wpi (Figure 3C).

TCBZ-induced variations in the test antigens were measured by IgG positivity following anthelmintic administration at 12 wpi, with changes to IgG patterns due to the induction and half-life of IgG additionally considered, both nominally three weeks in duration. As a result of these prerequisites, reduced or terminated (TCBZ-S phenotype) and continued or augmented (TCBZ-R phenotype) biomarker abundances were expected as a reflection of their involvement during TCBZ-induced killing (causing IgG induction) or survival (causing IgG reduction and triggering IgG titre decrease) phenotypes, respectively. The negativity observed during TCBZ-S fluke infection against rFhTPI, except during fluke patency expected at 11–12 wpi, indicates ablated FhTPI abundance post-treatment that fits the expected TCBZ-S phenotype (Figure 3C). Furthermore, anti-rFhTPI IgG positivity overlapping juvenile, adult, and TCBZ administration phases during TCBZ-R infection suggests persisting FhTPI production and immune exposure in the TCBZ-R versus TCBZ-S phenotype (Figure 3D).

## 3. Discussion

The crisis in fasciolosis management inspires the development of new diagnostic approaches, especially for the detection of TCBZ-R *F. hepatica* strains. Critical levels of TCBZ resistance are now reported worldwide [6,7] and are appearing at a rate that is set to increase alongside fluke incidence as a result of climate change [4,21,22]. As it stands, existing coprological, immunological, and molecular test options are unsuitable for on-field-based and point-of-care settings, which have long been an established practice for some other neglected tropical diseases [23], but only recently considered a goal for fasciolosis control [24,25]. On-farm monitoring of anthelmintic efficacy status will be a key component in regaining control over liver fluke infections via prolonging TCBZ shelf-life. To this end, our targeted molecular diagnostic pipeline tested two novel biomarkers of reported in vitro TCBZ-SO efficacy association [26] and confirmed in vivo immune signal exposure, providing a baseline upon which further field-based and point-of-care tests can be developed for infection and anthelmintic efficacy diagnoses.

**Figure A2:**
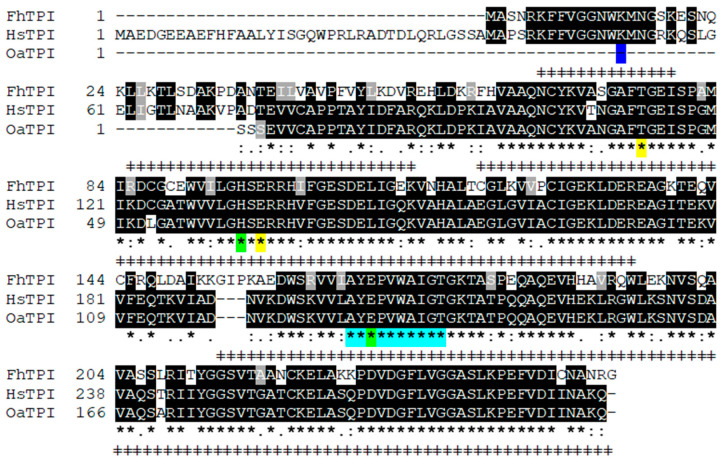
LC-MS/MS identification of FhTPI and comparison with host TPI proteins via sequence alignment. Sequence analysis of FhTPI is shown, including LC-MS/MS-identified residues (ǂ: GenBank, AGJ83762.1) based on 2-DE and duplicate 1-DE separated proteins (Figure 3C,D) that matched 86.5% FhTPI residues (GenBank, AGJ83762.1). Alignment of FhTPI with human (P60174) and ovine (W5P5W9) host proteins was conducted to identify sequence identity of 68.6% for both. Putative residues involved in structural and functional roles were indicated based on matching to HsTPI [17,18,19,20], including the TPI active site (FhTPI, A-167-T-176), and residues for dimerization (FhTPI, T-76, E-98), catalysis (FhTPI, K-14), or both processes (FhTPI, H-86, E-169), residues that were completely conserved between all three sequences here. Abbreviations: *, conserved; strongly semi-conserved; weakly semi-conserved.

rFh∆CRT and rFhTPI were recombinantly expressed and purified (Figure 1 and Figure 2), exhibiting analogous molecular properties as previously observed with native proteins ex vivo [26]. Bioinformatic analysis of FhTPI, following on from previous detailed biochemical evaluations [16], demonstrated the conservation of key residues pertaining to important TPI-specific structural and functional properties (Figure A2). Similar predictions and sequence comparisons for rFh∆CRT showed similarly high conservation of multiple residues of importance, and DNA sequencing and mass spectrometry data CRT confirmed a possible aspartate–asparagine residue substitution distinct from genome-derived sequences (Figure A1). Furthermore, investigation of potential antigenic cross-reactivity with human and ovine host orthologues found <70% similarity between all sequence comparison despite biologically important residue conservation, including sequence identities of 51.4–51.9% for FhCRT and 68.6% for rFhTPI.

Following the study by Morphew et al. [26], who identified several ES-derived proteins following in vitro sublethal (SL) or lethal (L) TCBZ-SO treatment, FhCRT recovery post-SL TCBZ-SO was putatively associated with survival in a virulence capacity, whereas L TCBZ-SO-induced treatment and FhTPI identification was presumed to be a biomarker of TCBZ susceptibility through an involuntary mechanism of internal and housekeeping protein exposure. Translation of these findings to an in vivo scenario was necessary to delineate the fluke’s complex molecular dynamics during parasite–host interactions and following treatment. To this end, anti-biomarker host IgG patterns against rFh∆CRT and rFhTPI were quantitatively determined from western hybridisations using sera of representative experimental TCBZ-S and TCBZ-R *F. hepatica* sheep infections (0–17 wpi), including TCBZ administration at 12 wpi. IgG positivity in the three weeks prior to 15 wpi in TCBZ-R sera against both biomarkers (Figure 3B,D) precluded definitive TCBZ-induced IgG recognition, as these could have resulted from continued antigen abundances or sustained IgG titres from previous exposures. However, TCBZ-R IgG against rFhΔCRT was negative between 9 and 13 wpi, but positive at 14–15 wpi, suggesting possible association of FhCRT with TCBZ challenge in the TCBZ-R phenotype (Figure 3B) and an ambiguous role following TCBZ treatment in the TCBZ-S infection phenotype (Figure 3A). Despite no evidence for TCBZ-S or TCBZ-R parasite-infected anti-antigen IgG specificity (Figure 3), anti-FhCRT IgG was detected at different intervals during TCBZ-S and TCBZ-R phenotype infections, including before and after TCBZ treatment, suggesting a multifaceted role of FhCRT towards housekeeping and/or virulence that is heightened or sustained during anthelmintic challenge. Conversely, anti-FhTPI IgG detection at multiple timepoints during infection with TCBZ-R compared with TCBZ-S parasites was indicative of heightened glycolytic activity or moonlighting roles by FhTPI, potentially supported by the negativity of anti-FhTPI IgG following TCBZ administration in the TCBZ-S infection sera.

FhCRT immunoreactivity was in keeping with previous strong CRT immunogenicity observations [27,28] and, particularly, recognition by TCBZ-R (Figure 3B) was in line with previous reports of CRT virulence association [28,29] and molecular phenotypes of drug-resistant cancer cells [30,31,32]. However, similarly to the influx of stress-response proteins during infection [33] and following TCBZ administration [26,34], FhCRT secretion during TCBZ-S infection, including after TCBZ at 15 wpi (Figure 3A), could be attributed to CRT’s nominal house-keeping functionalities. As a result of roles involving calcium homeostasis [35,36], glycoprotein and misfolded protein binding and chaperoning [37,38], and cellular recovery [37,38,39,40], CRT may have far-reaching direct and indirect roles, especially if the influence of FhCRT spans across the binding of factors with known roles in virulence, metabolic, or nutritional benefit and TCBZ response association, including cyclophilin A, glycolytic enzymes, and fatty acid binding proteins [26,34].

Similarly, different guises of glycolytic enzyme secretion under certain circumstances have been linked to pathogen virulence [41,42] as well as apoptosis [43], though TPI has so far only been linked to the latter process. It was thus expected that TPI would have a limited role and exposure during the TCBZ-R phenotype as per its anticipated internal, hidden antigen hallmarks and its overabundance in TBCZ-S fluke ES following in vitro L TCBZ-SO treatment [26]. However, as FhTPI recognition was more evident during TCBZ-R versus TCBZ-S fluke infection (Figure 3C,D), with anti-FhTPI IgG after TCBZ administration in the TCBZ-R group only, this suggests active secretion patterns irrespective of TCBZ challenge and presents as a potential biomarker of TCBZ-R fluke viability and TCBZ efficacy.

Molecular processes driving the observed TCBZ-S and TCBZ-R fluke infection and anthelmintic phenotypes, evidential FhCRT and FhTPI secretion in the lack of TCBZ administration, and FhTPI secretion following TCBZ administration by TCBZ-R flukes were intriguing. However, the diagnostic roles of FhCRT and FhTPI studied within the current TCBZ-S and TCBZ-R molecular profiles present some ambiguity, which invites further study to clarify their roles during *F. hepatica* infection towards virulence, nutrition, and/or xenobiotic metabolic processes. Moreover, and towards further diagnostic validation, future direct determinations of antigen abundance in host samples in response to anthelmintic application would be an ideal progression from this study, in order to provide representative and measurable changes in the TCBZ-S and TCBZ-R molecular phenotypes during infection and following TCBZ challenge. Together, these findings have identified multiple, descriptive elements of the changing proteomic molecular phenotype by TCBZ-S and TCBZ-R flukes during sheep infection and following TCBZ challenge, shown through the detection of anti-FhCRT and FhTPI IgG antibodies.

## 4. Materials and Methods

### 4.1. Cloning and Recombinant Protein Expression

The calreticulin gene sequence was identified in the *Fasciola hepatica* genome (WormBase ParaSite (WBPS) v14, parasite.wormbase.org; PRJEB25283: FhCRT, maker-scaffold10x_1445_pilon-snap-gene-0.69.mRNA-1 (WBPS v9: PRJEB6687, BN1106_s2673B000071.mRNA 1) for primer designs, including truncation of the N-terminal signal peptide (Fh∆CRT). cDNA was used for PCR (MyFi™, Bioline, UK) and amplicons were cloned using pGEM^®^-T Easy (Promega, UK) and alpha-select bronze efficiency cells (Bioline, UK) with ampicillin selection (100 μg/µL) (LB-agar, Melford, UK; LB broth, Sigma-Aldrich, UK), according to the manufacturer’s guidelines. Plasmids were extracted (ISOLATE II Plasmid Mini Kit, Bioline, UK) to introduce NdeI and NotI sites via PCR and cloned as before prior to restriction digestion (Thermofisher, UK) and ligation with complementarily-linearised pET-28B+ (Merck Millipore, Germany), as recommended by the manufacturers. Prepared expression plasmids *FhTPI*-pET-46 Ek/LIC [16], gifted by Professor David Timson (University of Brighton, UK), and *Fh∆CRT*-pET-28B+ were confirmed via triplicate Sanger sequencing (conducted in-house), including polyhistidine sequences at N- or C-termini (N: FhTPI, 5′-MAHHHHHHVDDDDK-3′; C: Fh∆CRT, 5′-AAALEHHHHHH-3′). Protein molecular weight and pI (accounting for polyhistidine tag) were predicted using ExPASy (web.expasy.org/compute_pi).

*Fh∆CRT*-pET-28B(+) was transformed into BL21(DE3)pLysS cells (Promega, UK) maintained with kanamycin (50 μg/µL) and *FhTPI*-pET-46 Ek/LIC was transformed into One Shot^®^ BL21(DE3) Star™ cells (Thermofisher, UK) maintained with ampicillin (100 μg/µL). For recombinant expression, liquid cultures were diluted (1:70) into fresh Luria-Bertani (LB) broth with appropriate antibiotic selection and grown until reaching 0.6–1.0 A_600_ cell density. Following induction of expression with isopropyl β-d-1–thiogalactopyranoside (IPTG) (0.1–1 mM), cultures were incubated at 37 °C and shaking at 150 revolutions per minute for five hours. Cells were collected by pelleting at 10,000× *g* at 4 °C for 10 min, and then resuspended in cold lysis buffer (0.1 M sodium phosphate, pH 7.4; 0.4 M sodium chloride; 5 mM magnesium chloride). For lysis, cells were subjected to three cycles of freeze–thawing in liquid nitrogen followed by three cycles of sonication and resting on ice for 15 s each. Crude soluble proteins were filtered with 0.22 μm polyethersulfone (Merck Millipore, Darmstadt, Germany) before polyhisitidine-tagged protein purification using immobilised metal ion chromatography (IMAC) with HIS-Select^®^ Nickel (FhTPI) or Cobalt (Fh∆CRT) (P6611 and H8162, Sigma-Aldrich, Gillingham, UK), with the procedure conducted as recommended by the manufacturer and with elutions tested using 50–500 mM imidazole. Purified IMAC fractions were filtered using Amicon^®^ Ultra 10K or 30K Centrifugal Filters (Merck, Gillingham, UK), as recommended by the manufacturer, and resuspended in ultrapure water (18.2 MΩ).

### 4.2. Proteomics and Western Hybridisation

Protein concentrations were calculated using the Bradford assay [44] and fractions were analysed by sodium dodecyl sulfate polyacrylamide gel electrophoresis (SDS PAGE) as follows, alongside the Amersham Low Molecular Weight SDS Calibration Kit (GE Healthcare Life Sciences, Amersham, UK; 14.4–97.0 kilodaltons, kDa) as recommended by the manufacturer. For 1-DE, 0.1–2 µg purified protein was used in 1–20 µL volume with SDS buffer (69.45 mM tris-HCl, pH 6.8; 11.1% (*v/v*) glycerol; 1% (*w*/*v*) SDS; 0.005% (*w*/*v*) bromophenol blue) including β–mercaptoethanol (100 mM) or dithiotreitol (DTT) (50 mM). For 2-DE procedures, 1–5 µg purified protein in 125 µL buffer A (8 M urea; 2% *w*/*v* CHAPS; 33 mM dithiotreitol (DTT); 0.5% *v*/*v* carrier ampholytes (Biolyte 3–10, Bio-Rad, Watford, UK)) was allowed to passively rehydrate 7 cm linear pH 3–10 IPG strips (Bio-Rad, UK) at room temperature overnight. Rehydrated strips were isoelectrically focused at 10,000–11,000 Vh using a Protean Mini IEF Cell (Bio-Rad, UK) to separate protein samples in the first dimension, then equilibrated in buffer B (50 mM tris-HCl; 6 M urea; 30% (*w*/*v*) glycerol; 2% (*w*/*v*) SDS) containing DTT (10 mg/mL) for 15 min, and then buffer B containing IAA (25 mg/mL) for a further 15 min. Electrophoresis was conducted at ambient temperature using the Protean Mini Tetra System (Bio-Rad, UK) in [1X] tris-glycine SDS buffer. Samples were aliquoted into wells (1-DE) or IPG strips were overlaid in molten agarose (0.5% (*w*/*v*) agarose; 0.125 M tris (pH 6.8); (100 mg/mL) bromophenol blue) (2-DE) prior to electrophoretic separation at 75 V through a stacking gel (4.5% acrylamide; 0.5 M tris, pH 6.8; 0.4% (*w*/*v*) SDS), followed by 150 V through the resolving gel (12.5% acrylamide; 1.5 M tris, pH 8.5; 0.4% (*w*/*v*) SDS) as required. Gels were fixed (10% (*v/v*) acetic acid; 40% (*v/v*) ethanol) and stained with Coomassie™ blue (PhastGel Blue R, Amersham Biosciences, Amersham, UK) with 10% (*v/v*) acetic acid and 30% (*v/v*) methanol or Coomassie™ blue solution (QC Colloidal Coomassie™, Bio-Rad), and then destained in 1% (*v/v*) acetic acid as required. Images were taken using a Typhoon™ FLA 9500 (GE Healthcare Life Sciences, Amersham, UK) using the digitization system for Coomassie™ Blue staining or using a Bio-Rad GS-800™ calibrated densitometer (Bio-Rad, UK) alongside Quantity One^®^ software (v4.6.9). Protein molecular weights were calibrated against the Amersham Low Molecular Weight SDS Calibration Kit using ImageQuant TL imaging analysis software (v8.1, GE Healthcare Life Sciences, Amersham, UK).

For protein identification, in-gel tryptic digestion was conducted prior to analysis by liquid chromatography tandem mass spectrometry according to the method previously described by Davis et al. [45], including duplicate samples for 1-DE-separated mono-morphic rFhTPI monomer and dimer bands in addition to negative gel piece controls for 1-DE and 2-DE sample preparation methods. Data files were submitted to a MASCOT MS/MS ions search (Matrix Science) against the *F. hepatica* genome (PRJEB6687, WBPS v9; cross-referenced and matched to PRJEB25283, WBPS v14) with search parameters as previously described [45], except allowing for 2+, 3+, or 4+ charged peptide residues and including a decoy search with each submission. The mass spectrometry proteomics data were deposited to the ProteomeXchange Consortium via the PRIDE [46] partner repository with the dataset identifier PXD017848 (DOI:10.6019/PXD017848), and details of sample nomenclature are available in the Supplementary Material (Table S1). Where required, *F. hepatica* biomarker DNA and protein sequences of interest identified from DNA sequencing and mass spectrometry, as well as host protein orthologs, were aligned and annotated using BioEdit (v7.0.5.3, [47]), Clustal Omega ([48]), and BOXSHADE (v3.2, ExPASy; embnet.vital-it.ch/software/BOX_form.html).

For western hybridisation procedures, 1-DE-separated samples (0.25 µg/lane) were transferred to nitrocellulose membrane (NCM 0.45 µm; GE Healthcare Life Sciences, Amersham, UK), which was confirmed by Amido Black staining, and membranes were prepared according to the method by Morphew et al. [11], with antibodies tested as follows. TCBZ-S and TCBZ-R *F. hepatica*-infected sera were used for primary antibody incubations, which were provided by Ridgeway Research Ltd. from three sheep infected (250 metacercariae dose) with one of three confirmed strains displaying phenotypes of TCBZ-S (Italian, Italy; Aberystwyth and Miskin, UK) or TCBZ-R (Kilmarnock, Penrith and Stornoway, UK). Parasites used for these infections were confirmed as TCBZ-R or TCBZ-S by Ridgeway Research Ltd. using faecal egg count reduction tests and in vitro TCBZ-SO exposure. The serum of all sheep was collected weekly during the course of infection from week 0 (uninfected) until week 17, including TCBZ treatment at 12 weeks post infection (wpi). All procedures performed on sheep (project licenses PPL 40/3593, P6D805744, and PA09B4E45) adhered to the United Kingdom Home Office Animals (Scientific Procedures) Act of 1986 as well as the European Union Animals Directive 2010/63/EU, and were approved by Ridgeway Research Ltd.’s Animal Welfare and Ethical Review Bodies. Sera were pooled per time point in each TCBZ-S/-R group to measure general immunoreactivity against the two *F. hepatica* antigens in these pilot tests, similarly to other studies investigating diagnostic efficacy and infection dynamics [49,50]. Primary serum antibodies were diluted to 1:1500 and incubated with membranes at room temperature for an hour prior to an incubation with anti-sheep secondary antibodies (1:30,000; anti-sheep IgG produced in donkey, A5187, Sigma-Aldrich, UK). Positive serum IgG binding was detected with 5–bromo-4–chloro-3–indolyl-phosphate (BCIP; Sigma-Aldrich, UK) and nitro blue tetrazolium (NBT; Thermo Scientific, UK) dissolved in alkaline phosphatase substrate solution (100 mM tris-HCl, pH 9.5; 100 mM NaCl; 5 mM MgCl_2_), until stopping in ultrapure water (18.2 MΩ), and using anti-histidine tag antibody (SAB1306082, Sigma-Aldrich, UK) (data not shown). Images of blots were taken using a Bio-Rad GS-800™ calibrated densitometer (Bio-Rad, UK) using default settings for alkaline phosphatase and exported via Quantity One^®^ software (v4.6.9). Positivity was ascertained following band quantification using ImageQuant TL imaging analysis software (v8.1), whereby band intensity was calculated following the subtraction of 0 wpi values from each wpi value, and data were summarised from duplicate western hybridisations, whereby values that were negative, or negative and positive in both replicates, were considered negative.

## 5. Conclusions

Our findings from immunologic testing with experimental infection sera revealed new evidence of the dynamic changes in the molecular proteome of infecting *Fasciola hepatica*, identified through IgG immunoreactivity. There were not only distinct differences over the course of infection (0–17 weeks) pertaining to test antigens rFh∆CRT and FhTPI, but further distinguishable IgG recognition patterns between TCBZ-S or TCBZ-R *F. hepatica* parasite infection and following TCBZ administration at 12 wpi. Specifically, evidence for in vivo FhCRT and FhTPI exposure was demonstrated during both TCBZ-S/R parasite infections in spite of TCBZ challenge and prior to anthelmintic administration, identifying these antigens as intriguing components of the molecular proteome that have eluded diagnostic research focus until now. Thus, recombinant and endogenous FhCRT and FhTPI possess diagnostic potential and, with test development, could permit differentiation between TCBZ-S and TCBZ-R liver fluke populations.

## Supplementary Materials

The following are available online. Table S1: Detailed nomenclature of rFh∆CRT and rFhTPI mass spectrometry samples, submitted using PRIDE [46] to the ProteomeXchange Consortium (dataset identifier: PXD017848, DOI:10.6019/PXD017848).
