# Supplementary material for: Pilot Evaluation of Two Fasciola hepatica Biomarkers for Supporting Triclabendazole (TCBZ) Efficacy Diagnostics
Source: Molecules. 2020 Jul 30;25(15):3477. doi: 10.3390/molecules25153477 (PMC7435721; doi:10.3390/molecules25153477)
Supplement: Supplementary file 1 [file molecules-25-03477-s001.pdf]

molecules

Supplementary Material

Pilot Evaluation of Two *Fasciola hepatica* Biomarkers for Supporting Triclabendazole (TCBZ) Efficacy Diagnostics

Clare F. Collett [1,*], Russell M. Morphew [1], David Timson [2], Helen C. Phillips [1], and Peter M. Brophy [1]

[1] Institute of Biological, Environmental and Rural Sciences, Aberystwyth University, Aberystwyth SY23 3DA, UK; rom@aber.ac.uk (R.M.M.); hcp5@aber.ac.uk (H.C.P.); pmb@aber.ac.uk (P.M.B.)
[2] School of Pharmacy and Biomolecular Sciences, University of Brighton, Brighton BN2 4GJ, UK; d.timson@brighton.ac.uk
* Correspondence: clare.collett@bristol.ac.uk

Table 1. Detailed nomenclature of rFhΔCRT and rFhTPI mass spectrometry samples, submitted using PRIDE [46] to the ProteomeXchange Consortium (dataset identifier: PXD017848, DOI:10.6019/PXD017848).

Sample	PRIDE File Name	File Type	Data Category
**rFhCRT 1-DE Sample A**	180814-1DSample8.dat	dat	Raw
	180814-Sample8.mgf	mgf	Peak
	180814-Sample8.pride.mgf.gz	pride.mgf	Peak
	180814-1DSample8_DECOY_[Fh.CRT_1-DE-FT_P0.01].mzid.gz	mzIDENTL	Result
	180814-1DSample8_DECOY_[Fh.CRT_1-DE-FT_P0.01].pride.mztab.gz	pride.mztab	Other
**rFhCRT 1-DE Sample B**	180814-1DSample9.dat	dat	Raw
	180814-Sample9.mgf	mgf	Peak
	180814-Sample9.pride.mgf.gz	pride.mgf	Peak
	180814-1DSample9_DECOY_[Fh.CRT_1-DE-10_P0.01].mzid.gz	mzIDENTL	Result
	180814-1DSample9_DECOY_[Fh.CRT_1-DE-10_P0.01].pride.mztab.gz	pride.mztab	Other
**rFhCRT 2-DE spot 1**	180814-2DSample1.dat	dat	Raw
	180814-2DSample1.mgf	mgf	Peak
	180814-2DSample1.pride.mgf.gz	pride.mgf	Peak
	180814-2DSample1_DECOY_[Fh.CRT_2-DE-spot1_P0.01].mzid.gz	mzIDENTL	Result
	180814-2DSample1_DECOY_[Fh.CRT_2-DE-spot1_P0.01].pride.mztab.gz	pride.mztab	Other
**rFhCRT 2-DE spot 2**	180814-2DSample2.dat	dat	Raw
	180814-2DSample2.mgf	mgf	Peak
	180814-2DSample2.pride.mgf.gz	pride.mgf	Peak
	180814-2DSample2_DECOY_[Fh.CRT_2-DE-spot2_P0.01].mzid.gz	mzIDENTL	Result
	180814-2DSample2_DECOY_[Fh.CRT_2-DE-spot2_P0.01].pride.mztab.gz	pride.mztab	Other

**rFhTPI 1-DE replicate 1**	180124-Sample10.dat	dat	Raw
	180124-Sample10.mgf	mgf	Peak
	180124-Sample10.pride.mgf.gz	pride.mgf	Peak
	180124-Sample10_DECOY_[FhTPI-1DE-30kDa_rep1_P0.01].mzid.gz	mzIDENTL	Result
	180124-Sample10_DECOY_[FhTPI-1DE-30kDa_rep1_P0.01].pride.mztab.gz	pride.mztab	Other
**rFhTPI 1-DE replicate 2**	180124-Sample12.dat	dat	Raw
	180124-Sample12.mgf	mgf	Peak
	180124-Sample12.pride.mgf.gz	pride.mgf	Peak
	180124-Sample12_DECOY_[FhTPI-1DE-30kDa_rep2_P0.01].mzid.gz	mzIDENTL	Result
	180124-Sample12_DECOY_[FhTPI-1DE-30kDa_rep2_P0.01].pride.mztab.gz	pride.mztab	Other
**rFhTPI$_2$ 1-DE replicate 1**	180124-Sample11.dat	dat	Raw
	180124-Sample11.mgf	mgf	Peak
	180124-Sample11.pride.mgf.gz	pride.mgf	Peak
	180124-Sample11_DECOY_[FhTPI-1DE-60kDa_rep1_P0.01].mzid.gz	mzIDENTL	Result
	180124-Sample11_DECOY_[FhTPI-1DE-60kDa_rep1_P0.01].pride.mztab.gz	pride.mztab	Other
**rFhTPI$_2$ 1-DE replicate 2**	180124-Sample13.dat	dat	Raw
	180124-Sample13.mgf	mgf	Peak
	180124-Sample13.pride.mgf.gz	pride.mgf	Peak
	180124-Sample13_DECOY_[FhTPI-1DE-60kDa_rep2_P0.01].mzid.gz	mzIDENTL	Result
	180124-Sample13_DECOY_[FhTPI-1DE-60kDa_rep2_P0.01].pride.mztab.gz	pride.mztab	Other
**rFhTPI 2-DE spot 1**	180124-Sample14.dat	dat	Raw
	180124-Sample14.mgf	mgf	Peak
	180124-Sample14.pride.mgf.gz	pride.mgf	Peak
	180124-Sample14_DECOY_[FhTPI-2-DE_spot1_P0.01].mzid.gz	mzIDENTL	Result
	180124-Sample14_DECOY_[FhTPI-2-DE_spot1_P0.01].pride.mztab.gz	pride.mztab	Other
**rFhTPI 2-DE spot 2**	180124-Sample15.dat	dat	Raw
	180124-Sample15.mgf	mgf	Peak
	180124-Sample15.pride.mgf.gz	pride.mgf	Peak
	180124-Sample15_DECOY_[FhTPI-2-DE_spot2_P0.01].mzid.gz	mzIDENTL	Result
	180124-Sample15_DECOY_[FhTPI-2-DE_spot2_P0.01].pride.mztab.gz	pride.mztab	Other
**rFhTPI 2-DE spot 3**	180124-Sample16.dat	dat	Raw
	180124-Sample16.mgf	mgf	Peak
	180124-Sample16.pride.mgf.gz	pride.mgf	Peak
	180124-Sample16_DECOY_[FhTPI-2-DE_spot3_P0.01].mzid.gz	mzIDENTL	Result
	180124-Sample16_DECOY_[FhTPI-2-DE_spot3_P0.01].pride.mztab.gz	pride.mztab	Other
**rFhTPI 2-DE spot 4**	180124-Sample17.dat	dat	Raw
	180124-Sample17.mgf	mgf	Peak
	180124-Sample17.pride.mgf.gz	pride.mgf	Peak

	180124-Sample17_DECOY_[FhTPI-2-DE_spot4_P0.01].mzid.gz	mzIDENTL	Result
	180124-Sample17_DECOY_[FhTPI-2-DE_spot4_P0.01].pride.mztab.gz	pride.mztab	Other
**rFhTPI 2-DE spot 5**	180124-Sample18.dat	dat	Raw
	180124-Sample18.mgf	mgf	Peak
	180124-Sample18.pride.mgf.gz	pride.mgf	Peak
	180124-Sample18_DECOY_[FhTPI-2-DE_spot5_P0.01].mzid.gz	mzIDENTL	Result
	180124-Sample18_DECOY_[FhTPI-2-DE_spot5_P0.01].pride.mztab.gz	pride.mztab	Other
**rFhTPI 2-DE spot 6**	180124-Sample19.dat	dat	Raw
	180124-Sample19.mgf	mgf	Peak
	180124-Sample19.pride.mgf.gz	pride.mgf	Peak
	180124-Sample19_DECOY_[FhTPI-2-DE_spot6_P0.01].mzid.gz	mzIDENTL	Result
	180124-Sample19_DECOY_[FhTPI-2-DE_spot6_P0.01].pride.mztab.gz	pride.mztab	Other
**Negative control [1-DE method, 180815 batch]**	180815-Sample50.dat	dat	Raw
	180815-Sample50.mgf	mgf	Peak
	180815-Sample50.pride.mgf.gz	pride.mgf	Peak
	[NegativeControl-blank_gel_piece-1-DE_protocol]180815-Sample50_DECOY_P0.01.mzid.gz	mzIDENTL	Result
**Negative control [2-DE method, 180814 batch]**	180814-2DSample11-negative_control.dat	dat	Raw
	180814-2DSample11.mgf	mgf	Peak
	180814-2DSample11.pride.mgf.gz	pride.mgf	Peak
	180814-2DSample11-negative_control.mzid.gz	mzIDENTL	Result
**Negative control [1-DE method, 180124 batch]**	180124-Sample27.dat	dat	Raw
	180124-Sample27.mgf	mgf	Peak
	180124-Sample27.pride.mgf.gz	pride.mgf	Peak
	[NegativeControl-blank_gel_piece-1-DE_protocol]180124-Sample27_DECOY_P0.01.mzid.gz	mzIDENTL	Result
